# Big fruits with tiny tepals: An unusual new species of Lauraceae from southwestern China

**DOI:** 10.3897/phytokeys.179.62050

**Published:** 2021-07-19

**Authors:** Zhi Yang, Wei-Yin Jin, Bing Liu, David Kay Ferguson, Yong Yang

**Affiliations:** 1 College of Biology and Environment, Nanjing Forestry University, 159 Longpan Road, Nanjing 210037, Jiangsu, China Institute of Botany, the Chinese Academy of Sciences Beijing China; 2 State Key Laboratory of Systematic and Evolutionary Botany, Institute of Botany, the Chinese Academy of Sciences, 20 Nanxincun, Xiangshan, Beijing 100093, China Tonghua Normal University Tonghua China; 3 Tonghua Normal University, 950 Yucai Road, Dongchang District, Tonghua City, Jilin 134000, China University of Vienna Vienna Austria; 4 University of Vienna, Department of Paleontology, 1090 Vienna, Austria Nanjing Forestry University Nanjing China

**Keywords:** Lauraceae, morphology, new species, *
Phoebe
*, phylogeny, taxonomy

## Abstract

We collected an unusual new plant of *Phoebe* (Lauraceae) from southeastern Yunnan, China, which possesses more or less oblong leaves, paniculate inflorescences with strictly opposite lateral cymes, trimerous flowers with 4-locular stamens, and large fruits with tiny, equal, persistent tepals. Our molecular phylogenetic study based on nrITS, *LEAFY* and plastid *mat*K sequences suggests that this species belongs to a clade of *Phoebe* including *P. puwenensis*, *P. megacalyx*, and *P. macrocarpa*. However, this species differs from the latter three species by subglabrous twigs, leaves and inflorescences (vs. pubescent twigs, leaves and inflorescences in the latter three species), larger fruits (5–8 cm long vs. 1–4 cm long in the latter three species), and smaller tepals (1–2.5 mm long vs. 5–15 mm long in the latter two species). As a result, *Phoebe jinpingensis* sp. nov. is described and illustrated here as new to science.

## Introduction

The Lauraceae are woody plants, except for the hemiparasitic climber genus *Cassytha* L. (Linnaeus 1753), and have more than 3,000 species mainly distributed in tropical regions (Rohwer 1993). This family is notorious for its complicated taxonomy. Many Lauraceae are large trees, which makes it difficult to collect specimens in the field. Good quality specimens are rarely represented in herbaria, many species being known from only one or a few imperfect specimens lacking floral or fruiting characters. The integration of morphological and molecular evidence represents a promising way to better understand the diversity of the tropical family Lauraceae.

*Phoebe* Nees (Nees von Esenbeck 1836) contains ca. 100 species that are distributed in tropical and subtropical Asia (van der Werff 2001; Wei and van der Werff 2008). Morphologically, *Phoebe* is well-defined and differs markedly from other Asian genera of the *Persea* group by the persistent and appressed tepals at the base of fruits (vs. deciduous or persistent and spreading tepals), e.g. *Alseodaphne* Nees (Wallich 1831), *Alseodaphnopsis* H.W.Li & J.Li (Mo et al. 2017), *Dehaasia* Blume (Blume 1837), *Machilus* Nees (Wallich 1831), *Nothaphoebe* Blume (Blume 1851).

Recently, we collected both flower and fruit materials of one notable tree species while conducting field investigations in southeastern Yunnan, China. Further morphological and molecular phylogenetic studies indicate that this species is a new species of *Phoebe*. We thus describe it here as new to science.

## Material and methods

### Morphology and Anatomy

We conducted field collections and morphological observations, obtained voucher specimens, preserved flowers in FAA, and dried leaf materials with silica gel. Photographs of vegetative and reproductive characters were taken with a Nikon camera (D700). The preserved flowers were dissected and observed, and photographs were taken under a stereo microscope (Leica S8APO).

### Phylogeny

To determine the systematic position of our new species, we conducted a phylogenetic study using nrITS, *LEAFY* and plastid *mat*K sequences. This phylogeny included 40 species from five genera of the *Persea* group (*Alseodaphne*, *Alseodaphnopsis*, *Dehaasia*, *Machilus* and *Phoebe*). *Litsea acuminata* (Blume) Sa. Kurata (Kurata 1968) and *Litsea akoensis* Hayata (Hayata 1911) were selected as the outgroup. Markers used in this phylogenetic study were either sequenced in this study or obtained from the GenBank (https://www.ncbi.nlm.nih.gov/genbank/). Vouchers and accession numbers of sequences are listed in Table 1.

**Table 1.** Sequences obtained in this study and their vouchers. All voucher specimens are deposited in PE.

Species	ITS	*LEAFY*	*mat*K	Voucher specimen	Locality
*Alseodaphne huanglianshanensis* H.W.Li & Y.M.Shui	MW826229	MW849576	MW849603	Bing Liu 2447	China, Yunnan
*Alseodaphnopsis andersonii* (King ex Hook.f.) H.W.Li & J.Li	MW826228	MW849575	MW849602	Bing Liu 1584	China, Yunnan
*Alseodaphnopsis hokouensis* (H.W.Li) H.W.Li & J.Li	MW826237	MW849583	MW849610	Bing Liu 2358	China, Yunnan
*Alseodaphnopsis petiolaris* (Meisn.) H.W.Li & J.Li	MW826234	MW849580	MW849607	Bing Liu 1266	China, Yunnan
*Alseodaphnopsis sichourensis* (H.W.Li) H.W.Li & J.Li	MW826235	MW849581	MW849608	Bing Liu 1980	China, Yunnan
*Alseodaphnopsis ximengensis* H.W.Li & J.Li	MW826236	MW849582	MW849609	Bing Liu 4726	China, Yunnan
*Dehaasia incrassata* (Jack) Kosterm.	MW826238	MW849585	MW849612	X.H.Jin IN004	Indonesia.
*Litsea acuminata* (Blume) Kurata	MW826239	MW849586	MW849613	Y.Yang, T.T.Sun & W.Y.Jin 2016110811	China, Taiwan
*Litsea akoensis* Hayata	MW826240	MW849587	MW849614	Y.Yang, T.T.Sun & W.Y.Jin 2016110810	China, Taiwan
*Machilus bonii* Lec.	MW826241	MW849588	MW849615	Bing Liu 2133	China, Yunnan
*Machilus breviflora* (Benth.) Hemsl.	MW826242	MW849589	MW849616	Bing Liu 1827	China, Guangdong
*Machilus melanophylla* H.W.Li	MW826243	MW849590	MW849617	Bing Liu 1950	China, Yunnan
*Machilus robusta* W.W.Sm.	MW826244	MW849591	MW849618	Bing Liu 1524	China, Yunnan
*Phoebe angustifolia* Meisn.	MW826246		MW849620	Bing Liu 1490	China, Yunnan
*Phoebe cavaleriei* (H.Lév.) Y.Yang & Bing Liu	MW826245		MW849619	Bing Liu 2215	China, Yunnan
*Phoebe chekiangensis* C.B.Shang	MW826247	MW849592	MW849621	Bing Liu 2232	China, Hubei
*Phoebe formosana* (Hayata) Hayata	MW826248	MW849593	MW849622	Y.Yang, T.T.Sun & W.Y.Jin 2016110836	China, Taiwan
*Phoebe jinpingensis* Liu B. 1477	MW826231	MW849584	MW849611	Bing Liu 1477	China, Yunnan
*Phoebe jinpingensis* Liu B. 2050	MW826232	MW849578	MW849605	Bing Liu 2050	China, Yunnan
*Phoebe jinpingensis* Liu B. 2052	MW826233	MW849579	MW849606	Bing Liu 2052	China, Yunnan
*Phoebe jinpingensis* Liu B. 2417	MW826230	MW849577	MW849604	Bing Liu 2417	China, Yunnan
*Phoebe lanceolata* (Wall. ex Nees) Nees	MW826249	MW849594	MW849623	Bing Liu 2532	China, Yunnan
*Phoebe macrocarpa* C.Y.Wu	MW826250	MW849595	MW849624	Bing Liu 1239	China, Yunnan
*Phoebe megacalyx* H.W.Li	MW826251	MW849596	MW849625	Bing Liu 1988	China, Yunnan
*Phoebe nanmu* (Oliv.) Gamble	MW826252	MW849597	MW849626	Bing Liu 2227	China, Yunnan
Phoebe neurantha var. brevifolia H.W.Li	MW826253	MW849598	MW849627	Bing Liu 2270	China, Yunnan
*Phoebe neurantha* (Hemsl.) Gamble	MW826254	MW849599	MW849628	Bing Liu 1570	China, Yunnan
*Phoebe puwenensis* W.C.Cheng	MW826255	MW849600	MW849629	Bing Liu 2242	China, Yunnan
*Phoebe sheareri* (Hemsl.) Gamble	MW826256	MW849601	MW849630	Bing Liu 2224	China, Hubei
*Phoebe tavoyana* (Meisn.) Hook.f.	MW826257	–	MW849631	Bing Liu 1822	China, Hainan

Total DNA was extracted from silica-gel-dried leaves using Tiangen Plant Genomic DNA kits. To amplify the nrITS, *LEAFY* and plastid *mat*K fragments, we followed Li et al. (2004, 2011b) and Sang et al. (1997) in the primers and the PCR amplification.

Sequences were edited with Sequencher 4.1.4 (Gene Codes Corporation, Ann Arbor, Michigan, USA), aligned using MAFFT 7 (Katoh and Standley 2013), and then manually adjusted using BioEdit 7.2.5 (Hall 1999). MEGA-X was used to compute variable and parsimony-informative sites (Kumar et al. 2018). Phylogenetic analyses were based on concatenated sequences in Phylosuite v1.2.2 (Zhang et al. 2020). The best-fit nucleotide substitution model for each sequence was determined by Modelfinder (Kalyaanamoorthy et al. 2017) in Phylosuite v1.2.2 based on BIC (Schwarz and Gideon 1978). Bayesian inferences (BI) based on the concatenated sequences were carried out with MrBayes (Ronquist et al. 2012) in Phylosuite v1.2.2 with the following designations: number of generations 2,000,000, sampling frequency 1000; a relative burnin of 25.0% for diagnostics. Maximum likelihood (ML) analyses were run with the IQtree (Nguyen et al. 2014) in Phylosuite v1.2.2 with 1000 bootstrap replicates.

### Figure treatments

The line drawing was done manually with a black ink pen. Illustrations and photos showing morphological characters were edited and merged in Adobe Photoshop CS2 ver. 9.0. Phylogenetic trees were browsed and adjusted in FigTree ver. 1.4.0 (Rambaut 2012) and then improved with Adobe Illustrator CS ver. 11.0.0. A distribution map was generated with ArcGis ver. 10.0 (ESRI, Redlands, CA, USA; http://www.esri.com).

### Red list assessment

Extinction risk was assessed using IUCN categories and criteria (IUCN 2012). Population data and area of occupancy were obtained/estimated according to our field investigations.

## Results

### Phoebe jinpingensis

Taxon classificationPlantaeColeopteraCerambycidae

Bing Liu, Y.Yang, W.Y.Jin & Zhi Yang sp. nov.

300D9704-7ACD-5B90-B9E9-2657AAC2C676

urn:lsid:ipni.org:names:77218352-1

Figs 1 , 2 , 3

#### Type.

China. Yunnan Province: Jinping County, Mengla, Tuomazhai, 22°37'N, 103°01'E, elev. 956 m, 8 Apr 2014, *B.Liu, Y.Yang, Q.W.Lin, L.Jiang & X.J.Li 2050* (***holotype***: PE!; ***isotypes***: PE!).

#### Diagnosis.

This species is similar to *P. macrocarpa* C.Y.Wu (Wu and Wang 1957) and *P. megacalyx* H.W.Li (Lee et al. 1979) in the large fruits over 3 cm in diam., but differs from the latter two species by the subglabrous leaves being more or less oblong-elliptic and the larger fruits having smaller tepals.

#### Description.

**Figure 1.**
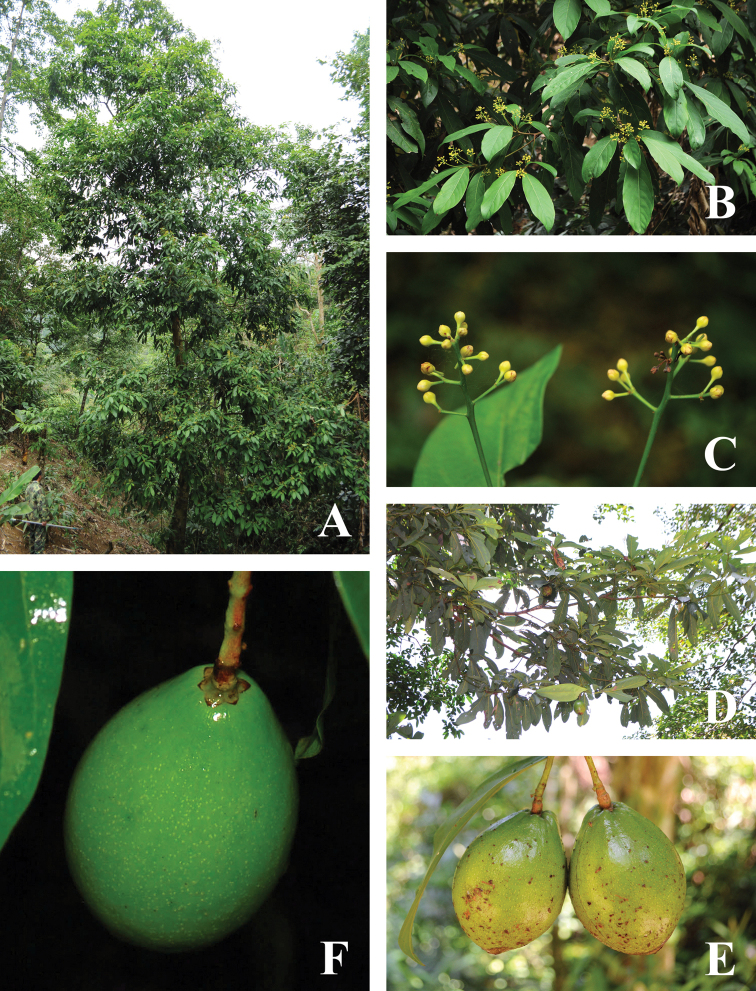
Morphology of *Phoebe jinpingensis* sp. nov. **A** habit of the tree **B** flowering branches **C** portion of a branch bearing inflorescences **D** fruiting branches **E** two fruits displaying swollen base and inconspicuous tepals **F** a fruit displaying the tiny tepals at the base.

Trees (Fig. 1A), up to 15 m tall, to 40 cm in DBH (diameter at breast height). Bark yellowish gray. Branchlets purplish, slender, longitudinally ridged, subglabrous. Leaves alternate, usually clustered at the apex of branchlets, thinly coriaceous to chartaceous, subglabrous, oblong to oblanceolate (Fig. 1B), 15–25 × 5–8 cm, apex acute to slightly acuminate, base acute, midvein impressed adaxially, and prominently elevated abaxially, lateral (secondary) veins 7–10 pairs, immersed adaxially and elevated abaxially, transverse minor (tertiary) veinlets connecting lateral veins visible; petioles 1.2–4 cm, subglabrous. Panicles slender, 4–9 cm long, subglabrous. Flowers yellowish (Fig. 1C). Tepals subequal, 2–2.4 mm long. Fertile stamens in three whorls; filaments of the first and second whorls 1–2 mm; anthers 4-locular, locules arranged in trapezoid shape; each filament of the 3^rd^ staminal whorl possessing two yellow cordate glands at its base. Staminodes sagittate. Fruits ellipsoid to obovoid, avocado-shaped, 5–8 cm long, and 3.5–5.2 cm in diam. (Fig. 1D–F); tepals persistent, equal, triangular to ovate, tiny, 2–2.5 mm long, clasping the fruit base (Fig. 1F), concealed and inconspicuous when fruit becoming swollen (Fig. 1E). Fruit peduncles not thickened.

**Figure 2.**
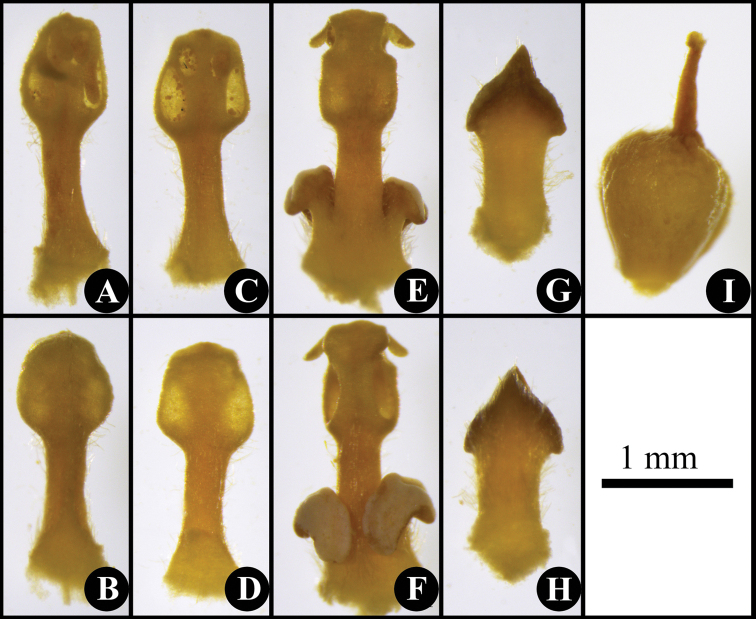
Anatomy of a flower of *Phoebe jinpingensis* sp. nov. showing morphology of stamens, staminodes, and pistil **A** stamens of the first staminal whorl, adaxial side **B** stamen of the first staminal whorl, abaxial side **C** stamen of the second staminal whorl, adaxial side **D** stamen of the second staminal whorl, abaxial side **E** stamen of the third staminal whorl, adaxial side **F** stamen of the third staminal whorl, abaxial side **G** staminode of the fourth staminal whorl, adaxial side **H** staminode of the fourth staminal whorl, abaxial side **I** pistil.

**Figure 3.**
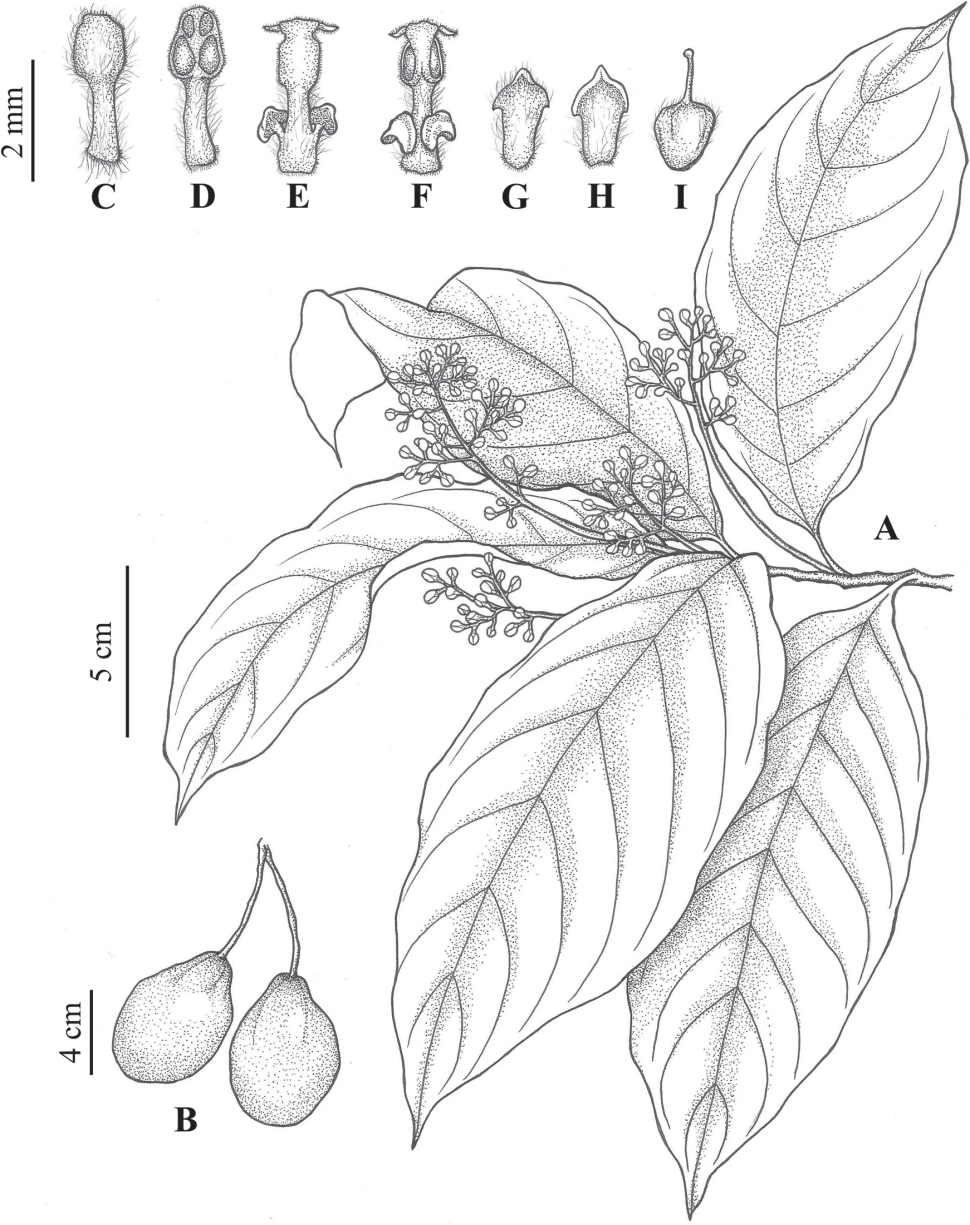
Line-drawing showing details of *Phoebe jinpingensis* sp. nov. **A** a flowering branch **B** fruits **C** fertile stamens of the first staminal whorl, abaxial side **D** fertile stamens of the first staminal whorl, adaxial side **E** fertile stamen of the third staminal whorl with the two glands at the base of the filaments, adaxial side **F** fertile stamen of the third staminal whorl with the two glands at the base of the filaments, abaxial side **G** staminode of the fourth staminal whorl showing pubescence, abaxial side **H** staminode of the fourth staminal whorl showing pubescence, adaxial side **I** pistil, with obovoid pubescent ovary and linear style.

#### Distribution.

**Figure 4.**
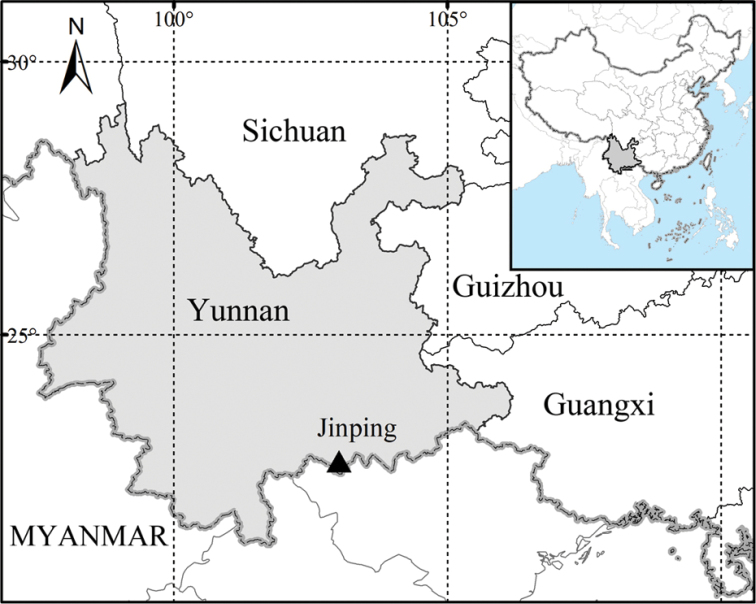
A map showing the distribution of *Phoebe jinpingensis* sp. nov.

So far, this species has only been found in southeastern Yunnan, China (Fig. 4).

#### Habitat.

This species occurs in montane evergreen forests at an altitudinal range of 900–980 m. It blooms in April, and the fruits mature from September to December.

#### Etymology.

The specific epithet ‘*jinpingensis*’ refers to the type locality “Jinping County”.

#### Additional specimens examined.

China. Yunnan Province: Jinping County, Mengla, Tuomazhai, 8 Apr 2014, fruit, *B.Liu, Y.Yang, Q.W.Lin, L.Jiang & X.J.Li 2052* (PE!); Jinping County, Mengla, Tuomazhai, 9 Oct 2011, fruit, *B.Liu 1477* (PE!); Jinping County, Mengla, Tuomazhai, 14 Sep 2014, fruit, *B.Liu, Y.Song, H.Lai & X.Yao 2417* (PE!).

#### Conservation.

There is only one population with 10 mature individuals occupying ca. 400 m^2^. Fewer than 10 juvenile individuals were found. All the individuals have not been protected in any nature reserve, and a rubber plantation exists nearby the population. Based on IUCN Red List Categories and Criteria (IUCN 2012), the new species is categorized as “Critically Endangered” (CR Blab (v); D).

##### Phylogeny

We finally obtained 30 sequences of nrITS, 27 sequences of *LEAFY* and 30 sequences of *mat*K (Table 2). The aligned nrITS sequences had 745 nucleotides including 149 variable sites among which 98 sites are parsimony-informative. The aligned *LEAF* sequences consisted of 896 nucleotides including 158 variable sites and 57 parsimony-informative. The aligned *mat*K sequences contained 763 nucleotides with 22 variable sites and 13 parsimony-informative sites. The concatenated matrix was 2404 nucleotides in length and included 329 variable sites and 168 parsimony-informative sites. The best-fit nucleotide substitution models for the three markers for both BI and ML analyses are listed in Table 3.

**Table 2.** The best-fit nucleotide substitution model of the three markers for both BI and ML analyses.

Analysis	nrITS	*LEAFY*	*mat*K
**BI**	HKY+F+I+G4	HKY+F+G4	HKY+F+I
**ML**	HKY+F+G4	HKY+F+G4	HKY+F

**Table 3.** Information of nrITS, *LEAFY*, *mat*K and concatenated sequences.

Item	nrITS	*LEAFY*	*mat*K	all
**Sequence length (nt)**	745	896	763	2404
**Variable (polymorphic) sites (nt)**	149	158	22	329
**Parsimony informative sites (nt)**	98	57	13	168

**Figure 5.**
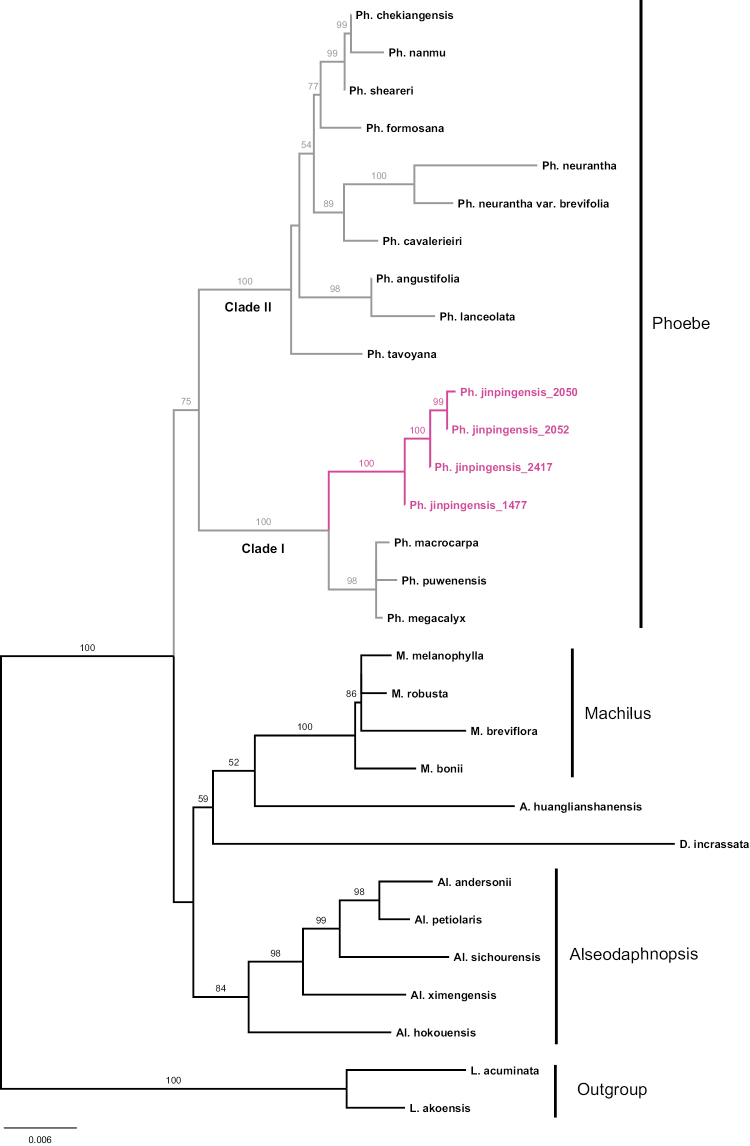
Maximum Likelihood tree based on nrITS, *LEAFY* and plastid *mat*K indicating the phylogenetic position of *Phoebe jinpingensis* sp. nov. Bootstrap support values (>50%) are shown above the branches.

**Figure 6.**
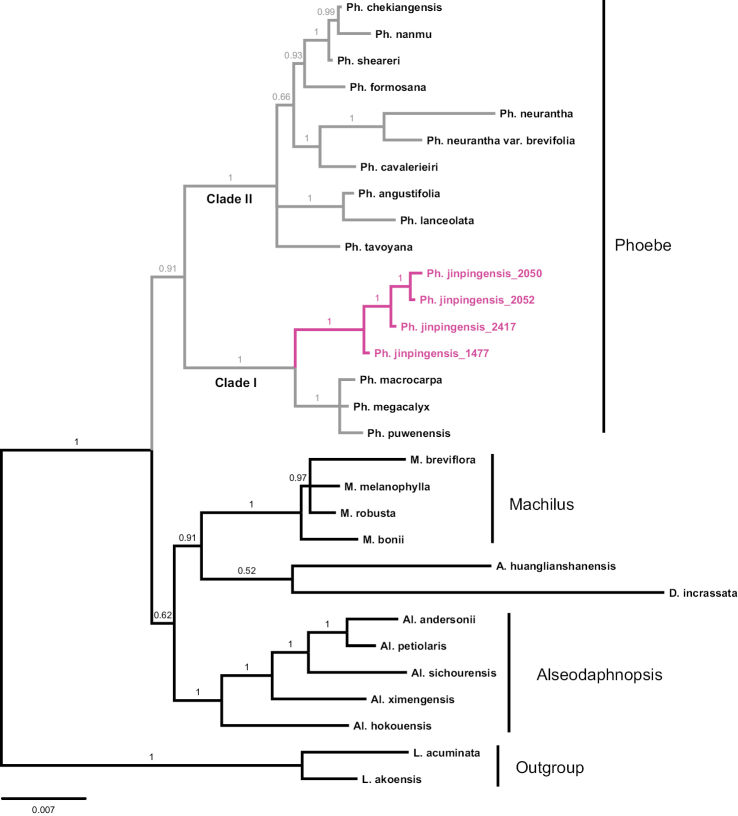
Bayesian tree based on nrITS, *LEAFY* and plastid *mat*K showing phylogenetic position of *Phoebe jinpingensis* sp. nov. Bayesian posterior probabilities (>0.50) are indicated above the branches.

Our phylogenetic study gave rise to BI and ML trees showing similar topology (Figs 5, 6). The genus *Phoebe* constituted a clade with moderate to high support (BS: 75, PP: 0.91), the two clades within the genus were robustly supported (BS: 100 and PP: 1). Clade I consisted of *P. puwenensis* W.C.Cheng (Cheng et al. 1963), *P. macrocarpa*, *P. megacalyx*, and *P. jinpingensis*. The four samples of our new species fell within a robust clade (BS: 100, PP: 1). Clade II includes *P. angustifolia* Meisn. (Meissner 1864), *P. cavaleriei* (H.Lév.) Y.Yang & Bing Liu (Yang and Liu 2015), *P. chekiangensis* C.B.Shang (Shang 1974), *P. formosana* (Hayata) Hayata (Hayata 1915), *P. lanceolata* (Wall. ex Nees) Nees (Nees von Esenbeck 1836), *P. nanmu* (Oliv.) Gamble (Sargent 1914), *P. neurantha* (Hemsl.) Gamble (Sargent 1914), *P. sheareri* (Hemsl.) Gamble (Sargent 1914), and *P. tavoyana* (Meisn.) Hook.f. (Hooker 1886).

*Machilus* was monophyletic (BS: 100 and PP: 1). *Alseodaphne huanglianshanensis* H.W.Li & Y.M.Shui (Li and Shui 2004) and *Dehaasia incrassata* (Jack) Kosterm. (Kostermans 1952) were close to *Machilus* (BS: 59%, PP: 0.91), but relationships among them were not resolved. *Alseodaphnopsis*, a recently established genus, constituted a monophyletic group which received high support (BS: 84%, PP: 1). Relationships among *Alseodaphnopsis*, *Phoebe*, and *Machilus*-*Dehaasia*-*Alseodaphne* were ambiguous.

## Discussion

Modern taxonomy is based on phylogeny. There is no phylogeny with full sampling of *Phoebe*, only a few molecular phylogenetic studies including partial sampling of the genus (Rohwer et al. 2009; Li et al. 2011a, b; Song et al. 2017). Frey (2015) stated that the genus *Phoebe* is polyphyletic but provided no evidence. Li et al. (2011a, b) reconstructed a phylogeny based on nrITS and nuclear *LEAFY* indicating that *Phoebe* as a monophyletic group receives low bootstrap value (<50%) but high posterior probability (Li et al. 2011a, b). Our new phylogenetic study using three markers reaches a similar conclusion. However, Song et al. (2017) reconstructed a phylogeny of *Phoebe* based on 15 highly variable regions of the chloroplast genome and found that the genus as a clade receives high support (BS: 100%; PP: 1.00). These phylogenetic studies consistently suggested that the traditional subdivision of the genus into two sections, i.e. sect. Phoebe and *Caniflorae* Meisn. (Meissner 1864), is unreasonable because some species of sect. Caniflorae are actually closer to species of sect. Phoebe. According to our phylogenetic study, the new species *Phoebe jinpingensis* belongs to the genus *Phoebe*, and falls within a robustly supported clade including *P. macrocarpa*, *P. megacalyx*, *P. puwenensis*, which were formerly ascribed to the sect. Caniflorae.

The genus *Phoebe* possesses persistent appressed tepals at the base of the fruits, whereas *Alseodaphne* and *Alseodaphnopsis* have fruits lacking persistent tepals (van der Werff 2001; Li et al. 2008; Mo et al. 2017). A few species formerly assigned to *Phoebe* with less indurate tepals slightly recurving at the apex and globose fruits were transferred to *Machilus* based on molecular phylogenetic studies (Li et al. 2011a). Traditional taxonomic studies have suggested that the genus *Phoebe* is characterized by fruits with persistent appressed tepals (van der Werff 2001; Li et al. 2008). Our new species, *P. jinpingensis*, clearly belongs to the genus *Phoebe*, because of its fruits having persistent and appressed tepals in fruits (Fig. 1E). Our phylogenetic study also confirmed that the new species belongs to *Phoebe*.

*Phoebe jinpingensis* is unusual in the genus *Phoebe*. First, the fruit is very large and avocado-shaped. Second, the tepals of *P. jinpingensis* are quite tiny (1–2.5 mm long) and concealed and not obvious in well-developed swollen fruits (Fig. 1E). Third, the leaf shape of *Phoebe* is usually oblanceolate, while our new species possesses oblong to oblanceolate leaves which is unusual in the genus and more similar to *Alseodaphne*/*Alseodaphnopsis*.

The genus *Phoebe* usually possesses small fruits ca. 1 cm, the largest fruits being seen in *P. megacalyx* (ca. 3.2 cm long) and *P. macrocarpa* (3.5–4.2 cm long) (Wei and van der Werff 2008). Our new species is most similar to *P. megacalyx* and *P. macrocarpa* in its large fruits, but differs from the latter two species by the subglabrous twigs, leaves and inflorescences (vs. pubescent or tomentose twigs, leaves, and inflorescences), the bigger fruits (5–8 cm long vs. 3–4 cm long in the latter two species), and the smaller tepals (1–2.5 mm long vs. >5 mm long in the latter two species). *Phoebe puwenensis* also belongs to the same clade as *P. jinpingensis* in our phylogenetic trees. Our new species differs from *P. puwenensis* in the subglabrous twigs and leaves (vs. tomentose twigs and leaves in the latter species) and the bigger fruits (5–8 cm long vs. 1–2 cm long in the latter species). For taxonomic purposes, we provide a new key to these closely related species of clade I of the genus *Phoebe*.

### Key to species of clade I of the genus *Phoebe*

**Table:** 

1	Leaves, inflorescences, and twigs subglabrous; fruits large, up to 8 cm long, having tiny tepals ca. 2–2.5 mm long	***P. jinpingensis***
–	Leaves, inflorescences, and twigs usually tomentose or pubescent; fruits smaller, usually ca. 1–4 cm, possessing bigger tepals 5 mm or longer	**2**
2	Fruits 3–4 cm long	**3**
–	Fruits 1–2 cm long	***P. puwenensis***
3	Leaves asymmetrical at the base; tepals woody, ca. 15 mm long	***P. megacalyx***
–	Leaves symmetrical at the base; tepals coriaceous, 5–6 mm long	***P. macrocarpa***

## Supplementary Material

XML Treatment for Phoebe jinpingensis

## Supplementary materials

Supplementary material 1 Figures S1–S8, Tables S1–S3 https://doi.org/10.3897/phytokeys.179.62050.suppl1 Data type Phylogenetic trees and tables Explanation note **Figures S1–S8.** Phylogenetic trees. Figures S1–S6. BI and ML trees of Asian species of the *Persea* group using single markers. Figures S7, S8. BI and ML trees of the Persea group using concatenated sequences of nrITS, *LEAFY* and *mat*K. **Table S1.** Sequences obtained from NCBI for phylogeny of the *Persea* group. **Table S2.** Substitution models of nrITS, *LEAFY* and *mat*K for phylogeny using separate markers. **Table S3.** A tabulated comparison of fruit size of *Phoebe* from China. https://binary.pensoft.net/file/567645This dataset is made available under the Open Database License (http://opendatacommons.org/licenses/odbl/1.0/). The Open Database License (ODbL) is a license agreement intended to allow users to freely share, modify, and use this Dataset while maintaining this same freedom for others, provided that the original source and author(s) are credited.Zhi Yang, Wei-Yin Jin, Bing Liu, David Kay Ferguson, Yong Yang
