# Supplementary material for: Big fruits with tiny tepals: An unusual new species of Lauraceae from southwestern China
Source: PhytoKeys. 2021 Jul 19;179:129–43. doi: 10.3897/phytokeys.179.62050 (PMC8313507; doi:10.3897/phytokeys.179.62050)
Supplement: Supplementary material 1 — Figures S1–S8, Tables S1–S3 [file phytokeys-179-129-s001.pdf]

Supplementary Materials

Figures

Fig. 1-8. Phylogenetic trees. 1-6, BI and ML trees of Asian species of the *Persea* group using single markers. **1**, BI tree using nrITS; **2**, ML tree using nrITS; **3**, BI tree using *LEAFY*; **4**, ML tree using *LEAFY*; **5**, BI tree using plastid *mat*K; **6**, ML tree using plastid *mat*K. **7-8**, BI and ML trees of the *Persea* group using concatenated sequences of nrITS, *LEAFY* and *mat*K. **7**, BI tree; **8**, ML tree.

Fig. 1

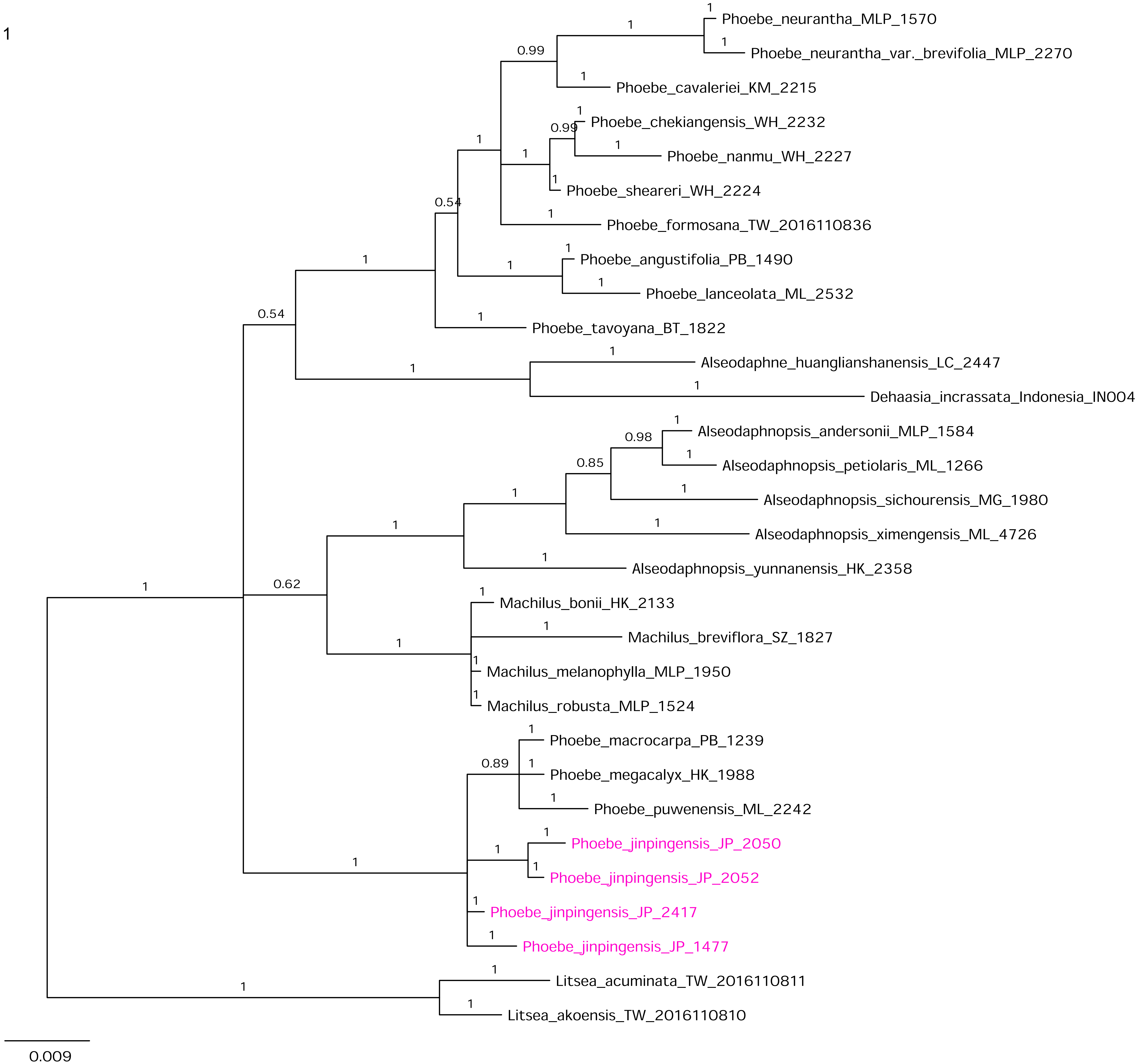
Phoebe_neurantha_MLP_1570
Phoebe_neurantha_var._brevifolia_MLP_2270
Phoebe_cavaleriei_KM_2215
Phoebe_chekiangensis_WH_2232
Phoebe_nanmu_WH_2227
Phoebe_sheareri_WH_2224
Phoebe_formosana_TW_2016110836
Phoebe_angustifolia_PB_1490
Phoebe_lanceolata_ML_2532
Phoebe_tavoyana_BT_1822
Alseodaphne_huanglianshanensis_LC_2447
Dehaasia_incrassata_Indonesia_IN004
Alseodaphnopsis_andersonii_MLP_1584
Alseodaphnopsis_petiolaris_ML_1266
Alseodaphnopsis_sichourensis_MG_1980
Alseodaphnopsis_ximengensis_ML_4726
Alseodaphnopsis_yunnanensis_HK_2358
Machilus_bonii_HK_2133
Machilus_breviflora_SZ_1827
Machilus_melanophylla_MLP_1950
Machilus_robusta_MLP_1524
Phoebe_macrocarpa_PB_1239
Phoebe_megacalyx_HK_1988
Phoebe_puwenensis_ML_2242
Phoebe_jinpingensis_JP_2050
Phoebe_jinpingensis_JP_2052
Phoebe_jinpingensis_JP_2417
Phoebe_jinpingensis_JP_1477
Litsea_acuminata_TW_2016110811
Litsea_akoensis_TW_2016110810
0.99
0.54
0.98
0.85
0.62
0.89
0.009

Fig. 2

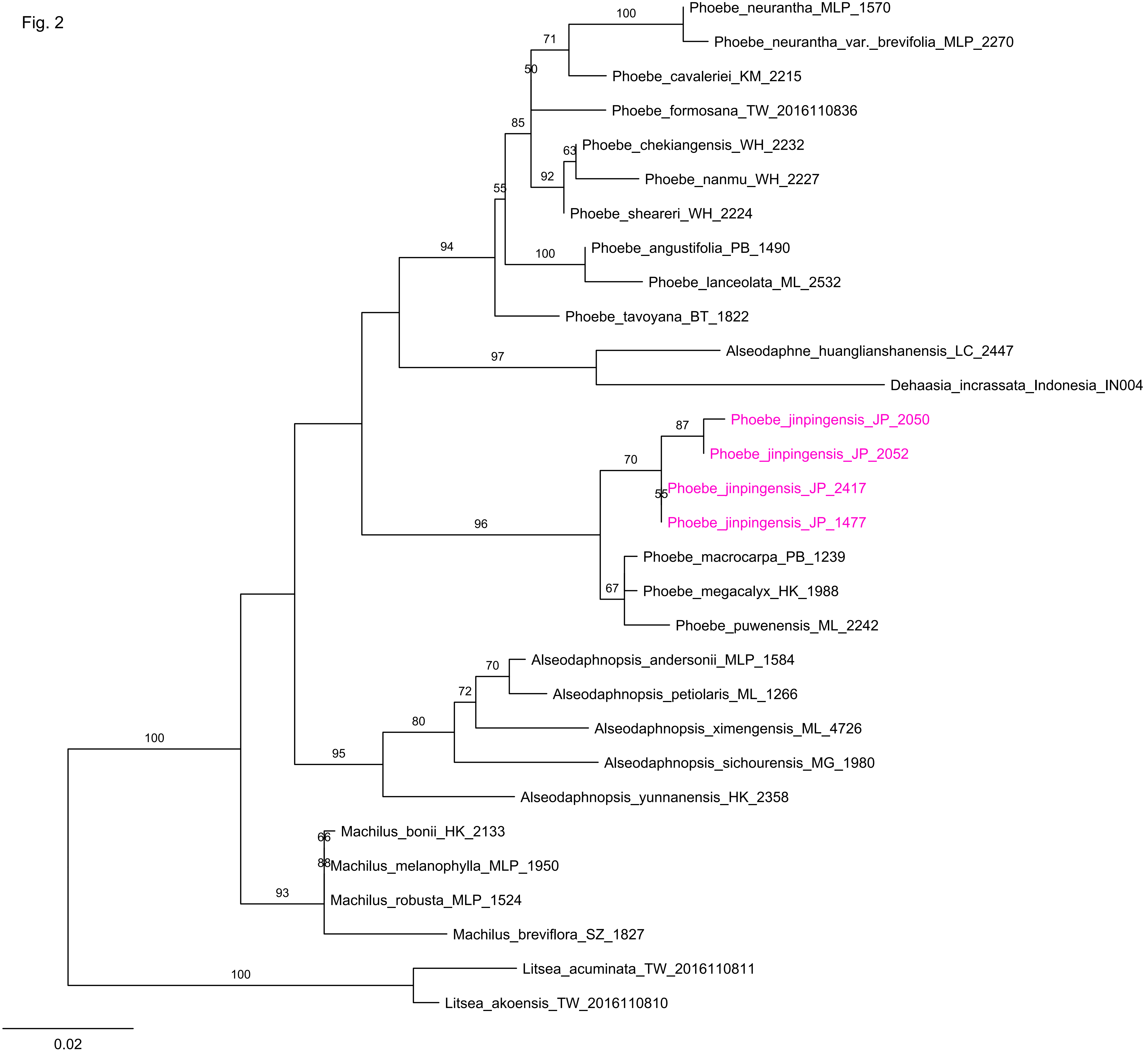

100
Phoebe_neurantha_MLP_1570
Phoebe_neurantha_var._brevifolia_MLP_2270
71
Phoebe_cavaleriei_KM_2215
50
Phoebe_formosana_TW_2016110836
85
63
Phoebe_chekiangensis_WH_2232
92
Phoebe_nanmu_WH_2227
55
Phoebe_sheareri_WH_2224
94
100
Phoebe_angustifolia_PB_1490
Phoebe_lanceolata_ML_2532
Phoebe_tavoyana_BT_1822
97
Alseodaphne_huanglianshanensis_LC_2447
Dehaasia_incrassata_Indonesia_IN004
87
Phoebe_jinpingensis_JP_2050
Phoebe_jinpingensis_JP_2052
70
55
Phoebe_jinpingensis_JP_2417
Phoebe_jinpingensis_JP_1477
96
Phoebe_macrocarpa_PB_1239
67
Phoebe_megacalyx_HK_1988
Phoebe_puwenensis_ML_2242
70
Alseodaphnopsis_andersonii_MLP_1584
72
Alseodaphnopsis_petiolaris_ML_1266
80
Alseodaphnopsis_ximengensis_ML_4726
100
95
Alseodaphnopsis_sichourensis_MG_1980
Alseodaphnopsis_yunnanensis_HK_2358
66
Machilus_bonii_HK_2133
88
Machilus_melanophylla_MLP_1950
93
Machilus_robusta_MLP_1524
Machilus_breviflora_SZ_1827
100
Litsea_acuminata_TW_2016110811
Litsea_akoensis_TW_2016110810
0.02

Fig. 3

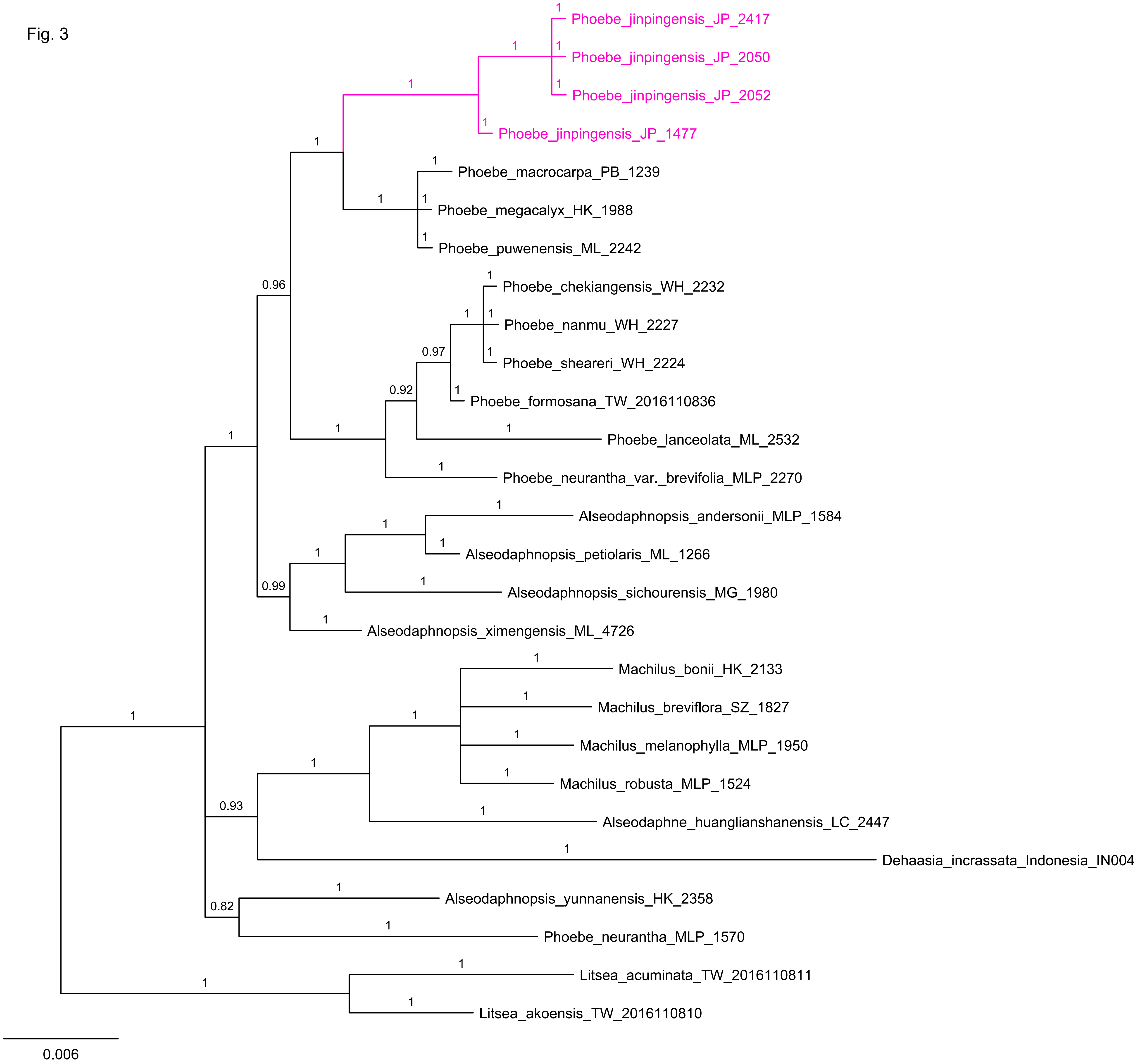

Phoebe_jinpingensis_JP_2417
Phoebe_jinpingensis_JP_2050
Phoebe_jinpingensis_JP_2052
Phoebe_jinpingensis_JP_1477
Phoebe_macrocarpa_PB_1239
Phoebe_megacalyx_HK_1988
Phoebe_puwenensis_ML_2242
Phoebe_chekiangensis_WH_2232
Phoebe_nanmu_WH_2227
Phoebe_sheareri_WH_2224
Phoebe_formosana_TW_2016110836
Phoebe_lanceolata_ML_2532
Phoebe_neurantha_var._brevifolia_MLP_2270
Alseodaphnopsis_andersonii_MLP_1584
Alseodaphnopsis_petiolaris_ML_1266
Alseodaphnopsis_sichourensis_MG_1980
Alseodaphnopsis_ximengensis_ML_4726
Machilus_bonii_HK_2133
Machilus_breviflora_SZ_1827
Machilus_melanophylla_MLP_1950
Machilus_robusta_MLP_1524
Alseodaphne_huanglianshanensis_LC_2447
Dehaasia_incrassata_Indonesia_IN004
Alseodaphnopsis_yunnanensis_HK_2358
Phoebe_neurantha_MLP_1570
Litsea_acuminata_TW_2016110811
Litsea_akoensis_TW_2016110810
0.96
0.97
0.92
0.99
0.93
0.82
0.006

Fig. 4

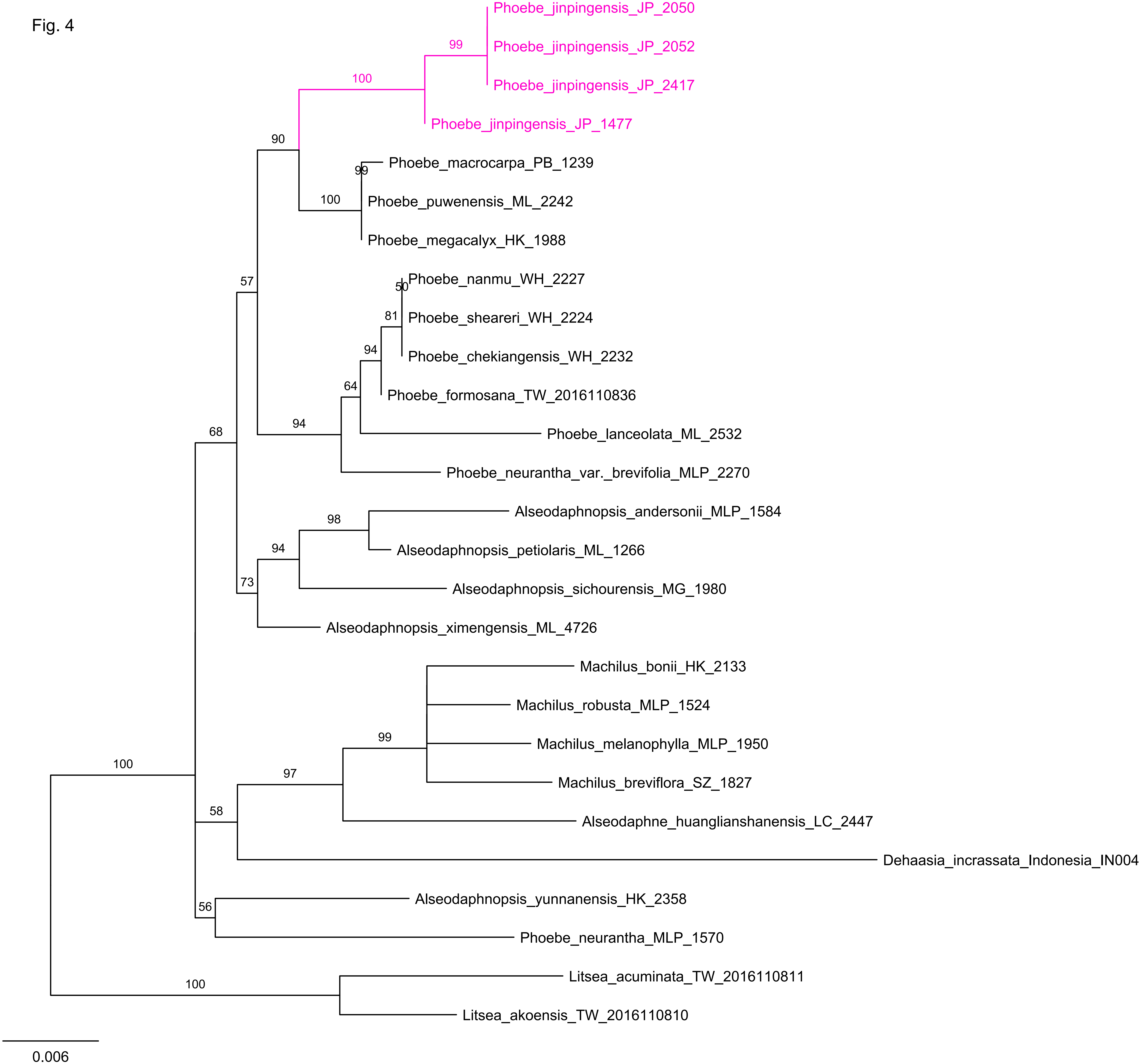
Phoebe_jinpingensis_JP_2050
Phoebe_jinpingensis_JP_2052
Phoebe_jinpingensis_JP_2417
Phoebe_jinpingensis_JP_1477
Phoebe_macrocarpa_PB_1239
Phoebe_puwenensis_ML_2242
Phoebe_megacalyx_HK_1988
Phoebe_nanmu_WH_2227
Phoebe_sheareri_WH_2224
Phoebe_chekiangensis_WH_2232
Phoebe_formosana_TW_2016110836
Phoebe_lanceolata_ML_2532
Phoebe_neurantha_var._brevifolia_MLP_2270
Alseodaphnopsis_andersonii_MLP_1584
Alseodaphnopsis_petiolaris_ML_1266
Alseodaphnopsis_sichourensis_MG_1980
Alseodaphnopsis_ximengensis_ML_4726
Machilus_bonii_HK_2133
Machilus_robusta_MLP_1524
Machilus_melanophylla_MLP_1950
Machilus_breviflora_SZ_1827
Alseodaphne_huanglianshanensis_LC_2447
Dehaasia_incrassata_Indonesia_IN004
Alseodaphnopsis_yunnanensis_HK_2358
Phoebe_neurantha_MLP_1570
Litsea_acuminata_TW_2016110811
Litsea_akoensis_TW_2016110810
99
100
90
99
100
50
81
94
64
57
94
68
98
94
73
99
97
100
58
56
100
0.006

Fig. 5

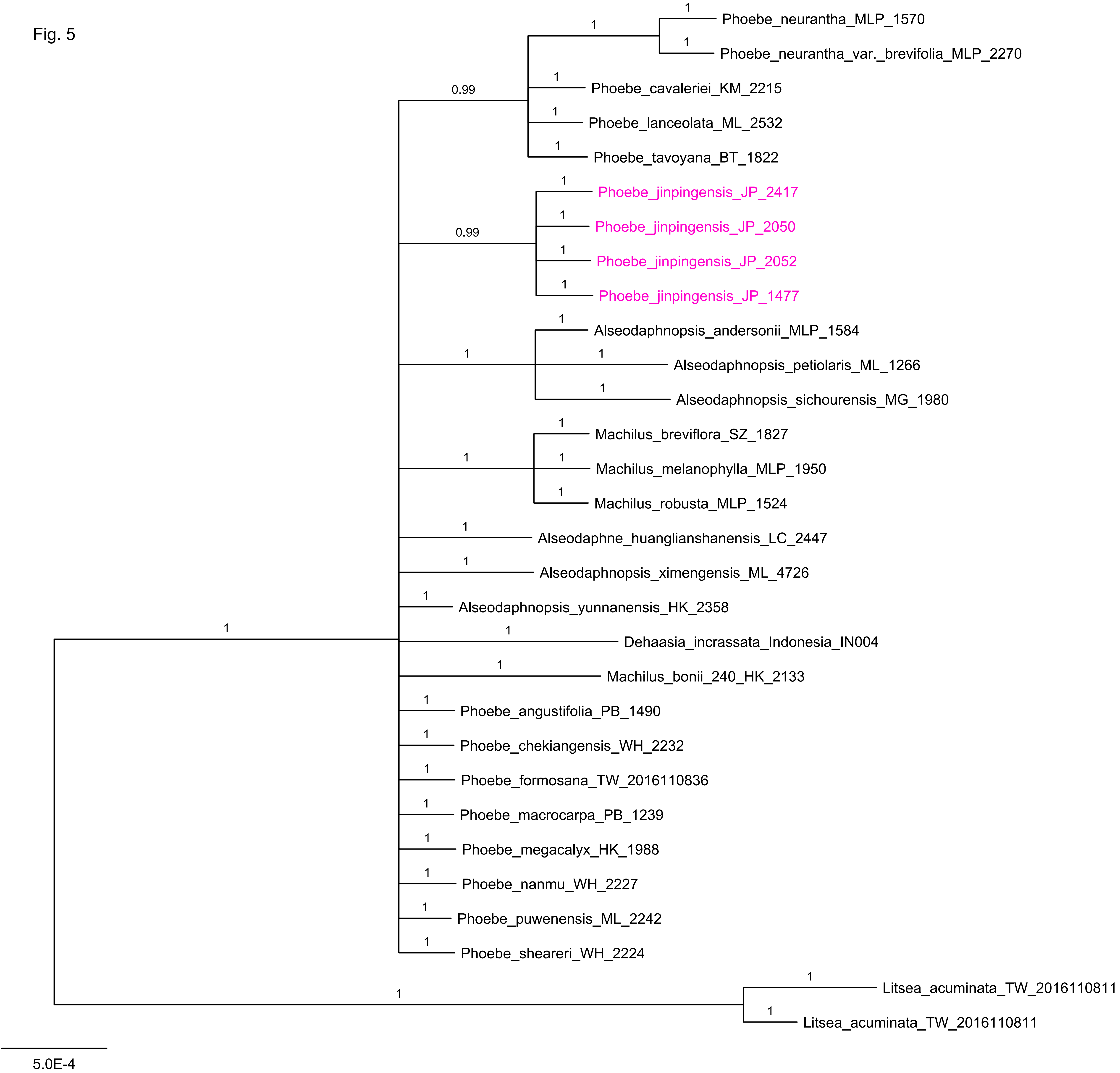

Phoebe_neurantha_MLP_1570
Phoebe_neurantha_var._brevifolia_MLP_2270
Phoebe_cavaleriei_KM_2215
Phoebe_lanceolata_ML_2532
Phoebe_tavoyana_BT_1822
Phoebe_jinpingensis_JP_2417
Phoebe_jinpingensis_JP_2050
Phoebe_jinpingensis_JP_2052
Phoebe_jinpingensis_JP_1477
Alseodaphnopsis_andersonii_MLP_1584
Alseodaphnopsis_petiolaris_ML_1266
Alseodaphnopsis_sichourensis_MG_1980
Machilus_breviflora_SZ_1827
Machilus_melanophylla_MLP_1950
Machilus_robusta_MLP_1524
Alseodaphne_huanglianshanensis_LC_2447
Alseodaphnopsis_ximengensis_ML_4726
Alseodaphnopsis_yunnanensis_HK_2358
Dehaasia_incrassata_Indonesia_IN004
Machilus_bonii_240_HK_2133
Phoebe_angustifolia_PB_1490
Phoebe_chekiangensis_WH_2232
Phoebe_formosana_TW_2016110836
Phoebe_macrocarpa_PB_1239
Phoebe_megacalyx_HK_1988
Phoebe_nanmu_WH_2227
Phoebe_puwenensis_ML_2242
Phoebe_sheareri_WH_2224
Litsea_acuminata_TW_2016110811
Litsea_acuminata_TW_2016110811
0.99
0.99
1
5.0E-4

Fig. 6

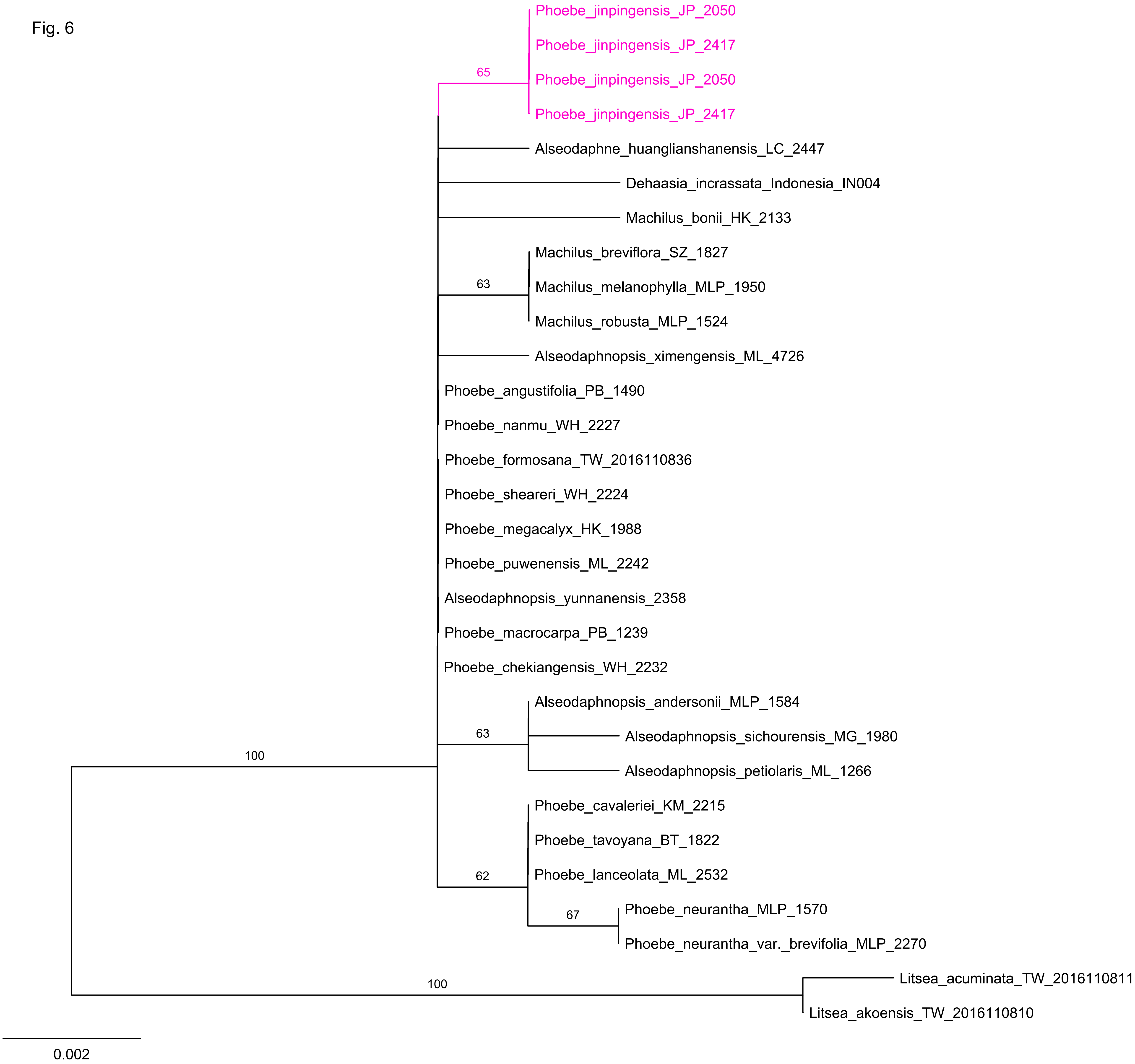
Phoebe_jinpingensis_JP_2050
Phoebe_jinpingensis_JP_2417
Phoebe_jinpingensis_JP_2050
Phoebe_jinpingensis_JP_2417
65
Alseodaphne_huanglianshanensis_LC_2447
Dehaasia_incrassata_Indonesia_IN004
Machilus_bonii_HK_2133
Machilus_breviflora_SZ_1827
Machilus_melanophylla_MLP_1950
Machilus_robusta_MLP_1524
63
Alseodaphnopsis_ximengensis_ML_4726
Phoebe_angustifolia_PB_1490
Phoebe_nanmu_WH_2227
Phoebe_formosana_TW_2016110836
Phoebe_sheareri_WH_2224
Phoebe_megacalyx_HK_1988
Phoebe_puwenensis_ML_2242
Alseodaphnopsis_yunnanensis_2358
Phoebe_macrocarpa_PB_1239
Phoebe_chekiangensis_WH_2232
Alseodaphnopsis_andersonii_MLP_1584
Alseodaphnopsis_sichourensis_MG_1980
Alseodaphnopsis_petiolaris_ML_1266
63
100
Phoebe_cavaleriei_KM_2215
Phoebe_tavoyana_BT_1822
Phoebe_lanceolata_ML_2532
62
Phoebe_neurantha_MLP_1570
Phoebe_neurantha_var._brevifolia_MLP_2270
67
Litsea_acuminata_TW_2016110811
Litsea_akoensis_TW_2016110810
100
0.002

Fig. 7

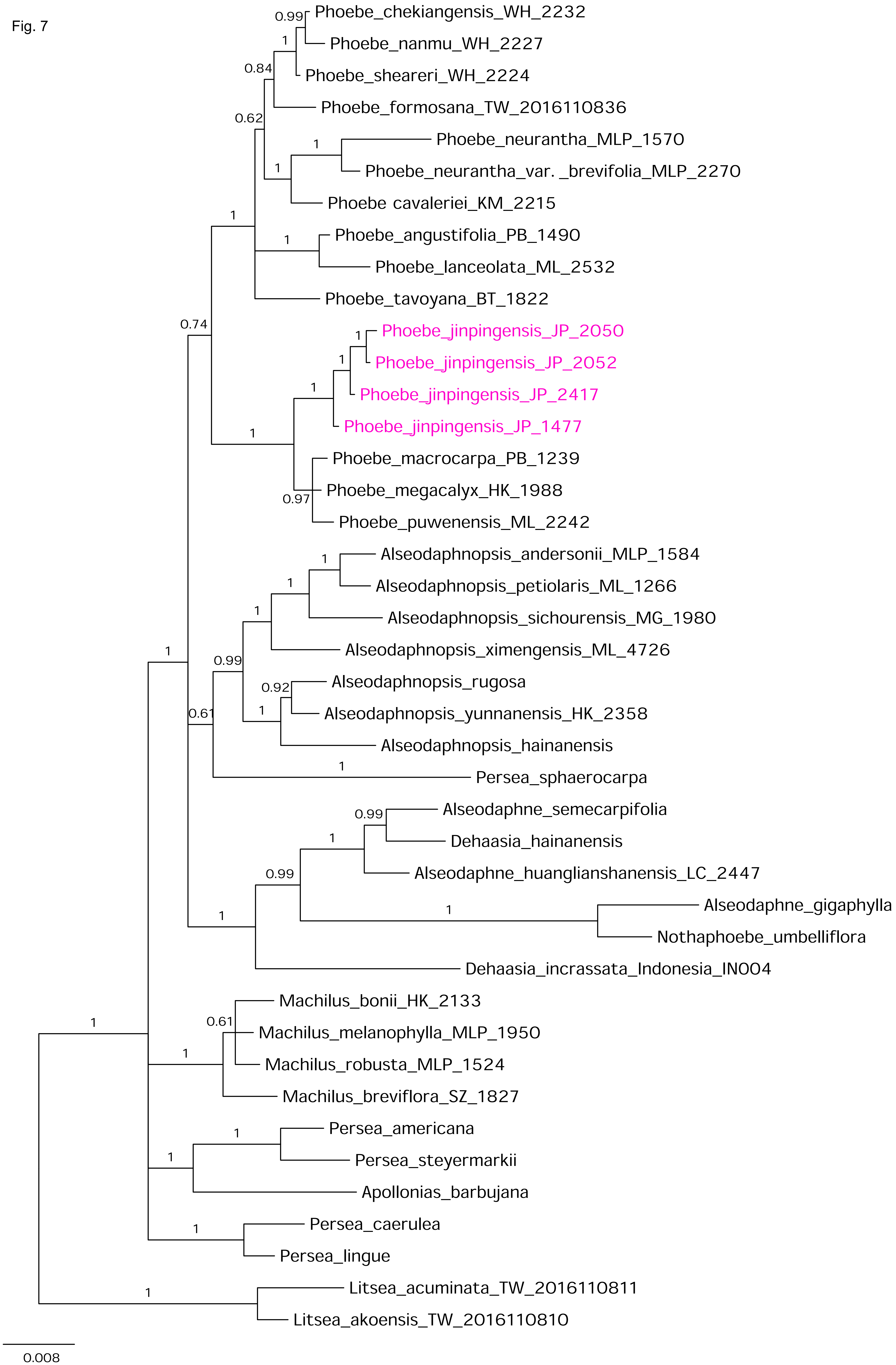

0.99
1
0.84
0.62
1
1
1
1
0.74
1
1
1
1
0.97
Phoebe_chekiangensis_WH_2232
Phoebe_nanmu_WH_2227
Phoebe_sheareri_WH_2224
Phoebe_formosana_TW_2016110836
Phoebe_neurantha_MLP_1570
Phoebe_neurantha_var. _brevifolia_MLP_2270
Phoebe cavaleriei_KM_2215
Phoebe_angustifolia_PB_1490
Phoebe_lanceolata_ML_2532
Phoebe_tavoyana_BT_1822
Phoebe_jinpingensis_JP_2050
Phoebe_jinpingensis_JP_2052
Phoebe_jinpingensis_JP_2417
Phoebe_jinpingensis_JP_1477
Phoebe_macrocarpa_PB_1239
Phoebe_megacalyx_HK_1988
Phoebe_puwenensis_ML_2242
1
1
1
0.99
0.92
1
0.61
1
Alseodaphnopsis_andersonii_MLP_1584
Alseodaphnopsis_petiolaris_ML_1266
Alseodaphnopsis_sichourensis_MG_1980
Alseodaphnopsis_ximengensis_ML_4726
Alseodaphnopsis_rugosa
Alseodaphnopsis_yunnanensis_HK_2358
Alseodaphnopsis_hainanensis
Persea_sphaerocarpa
0.99
1
0.99
1
1
Alseodaphne_semecarpifolia
Dehaasia_hainanensis
Alseodaphne_huanglianshanensis_LC_2447
Alseodaphne_gigaphylla
Nothaphoebe_umbelliflora
Dehaasia_incrassata_Indonesia_IN004
1
0.61
1
Machilus_bonii_HK_2133
Machilus_melanophylla_MLP_1950
Machilus_robusta_MLP_1524
Machilus_breviflora_SZ_1827
1
1
Persea_americana
Persea_steyermarkii
Apollonias_barbujana
1
Persea_caerulea
Persea_lingue
1
Litsea_acuminata_TW_2016110811
Litsea_akoensis_TW_2016110810
0.008

Fig. 8

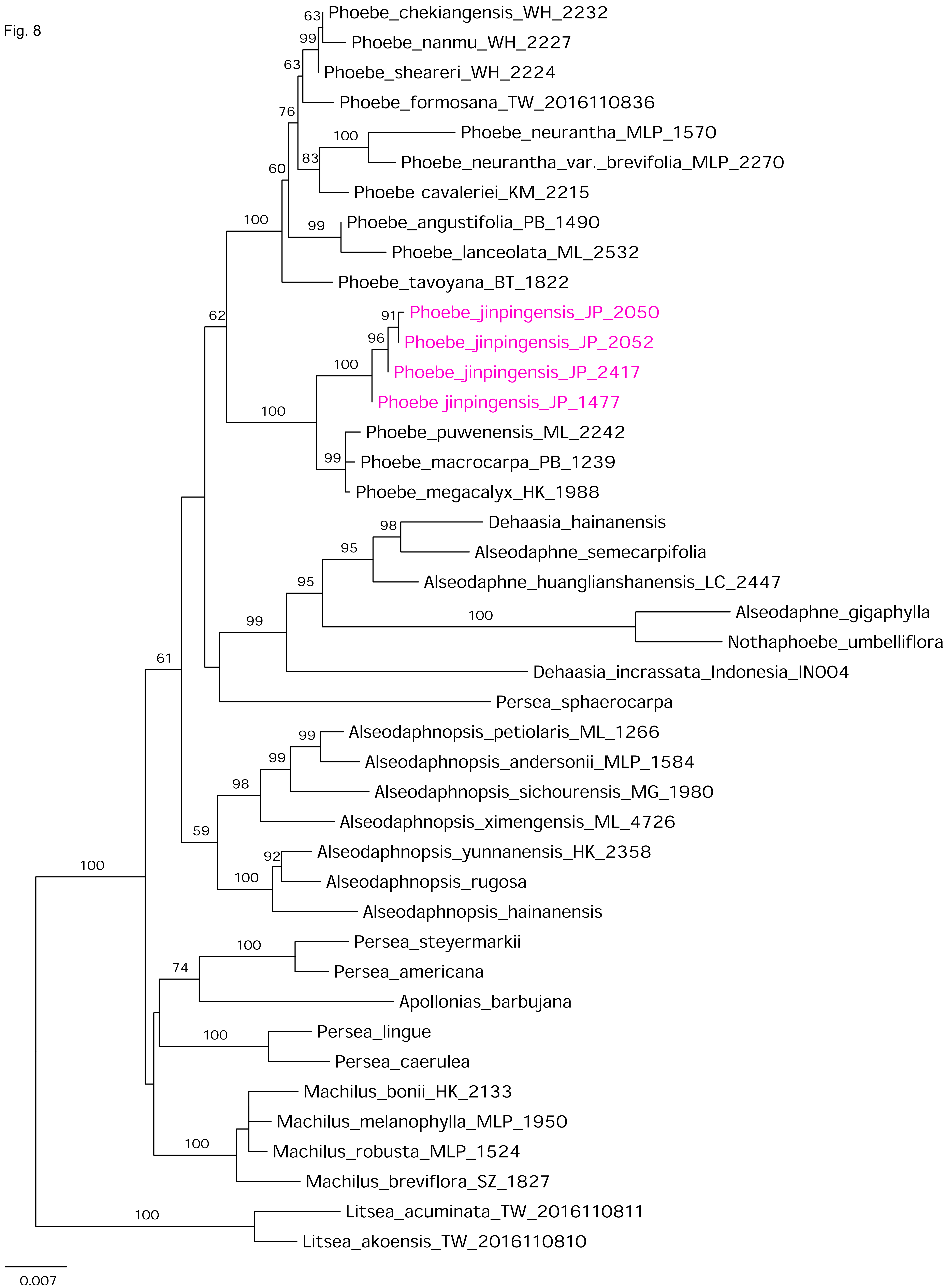
Phoebe_chekiangensis_WH_2232
Phoebe_nanmu_WH_2227
Phoebe_sheareri_WH_2224
Phoebe_formosana_TW_2016110836
Phoebe_neurantha_MLP_1570
Phoebe_neurantha_var._brevifolia_MLP_2270
Phoebe cavaleriei_KM_2215
Phoebe_angustifolia_PB_1490
Phoebe_lanceolata_ML_2532
Phoebe_tavoyana_BT_1822
Phoebe_jinpingensis_JP_2050
Phoebe_jinpingensis_JP_2052
Phoebe_jinpingensis_JP_2417
Phoebe jinpingensis_JP_1477
Phoebe_puwenensis_ML_2242
Phoebe_macrocarpa_PB_1239
Phoebe_megacalyx_HK_1988
Dehaasia_hainanensis
Alseodaphne_semecarpifolia
Alseodaphne_huanglianshanensis_LC_2447
Alseodaphne_gigaphylla
Nothaphoebe_umbelliflora
Dehaasia_incrassata_Indonesia_IN004
Persea_sphaerocarpa
Alseodaphnopsis_petiolaris_ML_1266
Alseodaphnopsis_andersonii_MLP_1584
Alseodaphnopsis_sichourensis_MG_1980
Alseodaphnopsis_ximengensis_ML_4726
Alseodaphnopsis_yunnanensis_HK_2358
Alseodaphnopsis_rugosa
Alseodaphnopsis_hainanensis
Persea_steyermarkii
Persea_americana
Apollonias_barbujana
Persea_lingue
Persea_caerulea
Machilus_bonii_HK_2133
Machilus_melanophylla_MLP_1950
Machilus_robusta_MLP_1524
Machilus_breviflora_SZ_1827
Litsea_acuminata_TW_2016110811
Litsea_akoensis_TW_2016110810
0.007

Supplementary Materials

Tables

Table SM-2. Substitution models of nrITS, *LEAFY* and *mat*K for phylogeny using separate markers (Supplementary Materials. 1-6).

**Analysis**	**nrITS**	***LEAFY***	***mat*K**
**BI**	HKY+F+G4	HKY+F+G4	HKY+F
**ML**	HKY+F+G4	HKY+F+G4	HKY+F

Table SM-1. Sequences obtained from NCBI for phylogeny of the *Persea* group (Supplementary Materials: 7 & 8).

**Taxon**	**nrITS**	***LEAFY***	***mat*K**
Alseodaphnopsis hainanensis (Merr.) H.W.Li et J.Li	FJ755440	HQ697005	-
Alseodaphnopsis rugosa (Merr. et Chun) H.W.Li et J.Li	HQ697183	HQ697011	-
Alseodaphne semecarpifolia Nees	HQ697184	HQ697014	-
Apollonias barbujana (Cav.) Bornm.	AY934889	HQ697021	-
Dehaasia hainanensis Kosterm.	FJ719308	HQ697026	-
Nothaphoebe gigaphylla (Kosterm.) Kosterm.	HQ697181	HQ697003	-
Nothaphoebe umbelliflora (Blume) Blume	HQ697191	HQ697088	-
Persea americana Mill.	AF272322	HQ697091	AJ247179
Persea caerulea (Ruiz et Pav.) Mez	FJ755436	HQ697099	EU153875
Persea lingue (Miers ex Bertero) Nees	HQ697196	HQ697102	JF966445
Persea sphaerocarpa (H.J.P.Winkl.) Kosterm.	FM957837	HQ697118	-
Persea steyermarkii C.K.Allen	HQ697200	HQ697120	JF966439

Table SM-3. A tabulated comparison of fruit size of *Phoebe* from China.

**Species**	**Fruit length (mm)**	**Fruit diam. (mm)**
Phoebe angustifolia Meisn.	9-12(14)	5-7
Phoebe bournei (Hemsl.) Yang	11-15	6-7
Phoebe brachythyrsa H.W.Li	11	7
Phoebe calcarea S.K.Lee et F.N.Wei	8-10	5-6
Phoebe cavaleriei (H.Lév.) Y.Yang et Bing Liu	12-14	12-14
Phoebe chekiangensis C.B.Shang	12-15	-
Phoebe chinensis Chun	10	10
Phoebe crassipedicella S.K.Lee et F.N.Wei	10	8
Phoebe faberi (Hemsl.) Chun	7-9	7-9
Phoebe formosana (Hayata) Hayata	8-9	-
Phoebe forrestii W.W.Smith	13	10
Phoebe glaucifolia S.K.Lee et F.N.Wei	10	5
Phoebe glaucophylla H.W.Li	18	10
Phoebe hainanensis Merr.	16-18	8
Phoebe hui Cheng ex Yang	12-14	6-9

Phoebe hunanensis Hand.-Mazz.	10-12	7
Phoebe hungmoensis S.K.Lee	10	5-6
Phoebe kwangsiensis H.Liu	-	-
Phoebe lanceolata (Wall. ex Nees) Nees	9-12	6-7
Phoebe legendrei Lec.	7-9	-
Phoebe lichuanensis S.K.Lee	-	-
Phoebe macrocarpa C.Y.Wu	38(42)	19-22
Phoebe megacalyx H.W.Li	32	18
Phoebe microphylla H.W.Li	4-5	1.8
Phoebe minutiflora H.W.Li	8	8
Phoebe motuonan S.Lee et F.N.Wei	20-22	15-16
Phoebe neurantha (Hemsl.) Gamble	10	7
Phoebe neuranthoides S.K.Lee et F.N.Wei	10-12	5-8
Phoebe nigrifolia S.K.Lee et F.N.Wei	10	8
Phoebe puwenensis W.C.Cheng	13	7
Phoebe rufescens H.W.Li	20-32	11-20
Phoebe sheareri (Hemsl.) Gamble	10	5-6
Phoebe tavoyana (Meisn.) Hook.f.	12	-
Phoebe yaiensis S.K.Lee	11-13	5-7
Phoebe yunnanensis H.W.Li	20-22	13
Phoebe zhennan S.Lee et F.N.Wei	11-14	6-7

Note: data of fruit size were extracted from *Flora of China*.
